# A Department of Public Health

**Published:** 1910-07

**Authors:** 


For Texas Medical Journal.

### A Department of Public Health.

OFFICE COMMITTEE OF ONE HUNDRED OF THE AMERICAN ASSOCIATION FOR THE ADVANCEMENT OF SCIENCE, ON NATIONAL HEALTH.

460 PROSPECT STREET, NEW HAVEN, CONN., June 14, 1910.

*To the Members of the Authors' League of the Committee of One Hundred:*

GENTLEMEN: I enclose a copy of our June bulletin, which will give you information about the "National League for Medical

Freedom," which is opposing our efforts to obtain a National Department of Health.

Of course, their opposition is ridiculous because the national government has not the power to interfere with the practice of medicine; because the framers of the bill (Senator Owen and his brother) did so independently of the American Medical Association; because the Committee of One Hundred is not the organ of the American Medical Association and never was, but started independently from the American Association for the Advancement of Science; and because members of our committee are, so far as known, in favor of "medical freedom."

This view I have expressed in my chapter on the medical profession in "National Vitality," a report of the National Conservation Commission to President Roosevelt.

In spite of this, thousands of foolish telegrams have been sent to Congressmen asking them to defeat the bill for a "doctors' trust."

A little ridicule will silence this opposition, yet the Congressmen are perhaps more apt than the public to be influenced by it, and it is important that all friends of the health movement should now show their strength.

I am writing to ask if you can not, on the basis of the above statement and the bulletin I am enclosing, say something against these people.

They seemed to be, at the time they were running the advertisement, spending $25,000 a day. It appeared all over the country. The lobbyists at Washington who opposed the Pure Food Bill were on hand to co-operate with this "League" in opposing the Owen bill.

The *New York Herald,* which resents the $25,000 fine they had to pay for their "personal" column advertising abortionists, etc., also resents the loss of the revenue from such sources, estimated at $200,000 a year, and has ever since been trying to whack the medical profession, and now is the one paper which is daily running articles against us.

A writer in New York was recently approached and offered a large sum of money to write articles to defeat the Owen bill. While buying newspaper space and hiring people to buy articles against the Owen bill, these same people are criticising the Committee of One Hundred for spending money, although we have never spent money in either of these ways, but only for sending out literature, correspondence, etc.

Mr. Allan Benson has written an article along these lines for the July number of *Pearson's Magazine.*

Hoping that you may be able to take a hand, I am,

Very sincerely yours,

IRVING FISHER.

P. S.—I also enclose a letter I have prepared for the press.

---

(Copy.)

460 PROSPECT STREET, NEW HAVEN, CONN., June 11, 1910.

*To the Editor Texas Medical Journal.*

DEAR SIR: Buying newspaper space appears to be the latest method of opposing a popular movement. Within a few weeks, three enormous advertisements, by a so-called "League for Medical Freedom," have appeared in newspapers throughout the country urging people to write their Congressmen in protest against the Owen bill for a National Department of Health. Much to the amusement of Congressmen, these advertisements have resulted in numerous telegrams, based on the absurd idea that the Owen bill aims to regulate the practice of medicine.

The result may be to aid, rather than hinder, the progress of the health movement. Already newspaper editorials—such as that which recently appeared in the *New York Times*—resent the buncombe of these advertisements.

But besides adding to the "gaiety of nations," this incident has created an appetite among the general public for more knowledge as to what this opposition signifies.

As President of the Committee of One Hundred on National Health, which was challenged in these advertisements, I wish to emphasize the following facts:

1. As the enclosed bulletin of the Committee of One Hundred indicates, the real strength of the opposition evidently comes from commercial interests, such as the quack medicine interests and others who have reason to fear the Pure Food and Drugs Act. While Christian Scientists and other "drugless" cults are denouncing drug doctors and denouncing a "medical trust" which does not exist, these cults are themselves playing into the hands of a drug trust which does exist.

2. Under our Constitution, the Federal government could not, if it would, regulate the practice of medicine.

3. The Owen bill contains no provision aiming to regulate the practice of medicine.

4. Section 8, in the Owen bill, the section authorizing the establishment of "chemical, biological, and other standards," has been eliminated entirely from the bill, although only a heated imagination could have construed this section as attempting to regulate the practice of medicine.

5. Senator Owen did not prepare his bill at the instance either of the American Medical Association or of the Committee of One Hundred on National Health. It is true, however, that the American Medical Association and the Committee of One Hundred heartily endorse the bill in preference to any of the other numerous health bills now before Congress.

6. The Committee of One Hundred on National Health is not a medical organization. It did not originate with the American Medical Association, but with the American Association for the Advancement of Science. It is allied with the American Medical Association only in the same sense that it is allied with labor organizations, farmer organizations, life insurance companies and various other agencies which are working to improve public health.

7. While opposed to fraudulent quackery, which is always imposing "fake" medicines and cure-alls on the public, the Committee of One Hundred on National Health is not devoted to any particular school of medicine to the exclusion of others. Curiously enough, it was a Christian Scientist who moved the appointment of the Committee of One Hundred. Many members of our Committee have been noted for their independence of conventional medicine, among them being ex-President Eliot of Harvard University, Mr. William H. Allen, Mr. Bok, Editor of the *Ladies' Home Journal*, Mr. Horace Fletcher, Dr. Luther H. Gulick, President G. Stanley Hall, Mrs. John B. Henderson, Dr. J. H. Kellogg, Mr. S. S. McClure of *McClure's Magazine*, Dr. Richard C. Newton who has called attention to some merits in Osteopathy and Mr. Nathan Strauss. I may add that in my report on "National Vitality" to President Roosevelt, I put myself on record as favoring "medical freedom." I can endorse almost all of the position on that subject taken by Mr. Flower.

8. A Department of Health in America like the Department of Health in Germany or anywhere else will have better things to do than regulate the practice of medicine. It will regulate the misbranding of foods and drugs (there's the rub!), the pollution of streams, the inspection of meats and quarantine and will obtain and distribute information in regard to health of human beings just as the Department of Agriculture does in regard to the health of hogs and cattle.

When the "League for Medical Freedom" suddenly appeared on the horizon, our movement had encountered substantially no opposition except among quacks and quack medicine proprietors. On the other hand, cur movement has the support of the President, of both political parties, as expressed in their platforms, of scientific, philanthropic, medical and labor organizations and the Granges, as well as of the life insurance companies, of Dr. H. W. Wiley of the Bureau of Chemistry and of General Walter Wyman of the Public Health and Marine Hospital Service. The general public will find it hard to believe that these endorsements, especially the hearty endorsement of the life insurance companies, can be in the interests of a "medical trust."

For further information, I would refer the reader to Bulletin 41 of the Committee of One Hundred about to be issued (address Drawer 45, New Haven, Conn.); to the Senate Reports (Nos. 1 and 2) on hearings on S. 6049 (address Senate document room); to House reports of hearings on health bills June 2-6 (address document room, House of Representatives); to Senator Owen's speeches (address Senator Owen) and to my report on "National Vitality" (Senate Document 419).

Very sincerely yours,

IRVING FISHER,

President Committee of One Hundred on National Health.

---

Subacute osteomyelitis at the mid-shaft of the tibia is often post-typhoidal.—*American Journal of Surgery.*

---

THERE was presented a bill to the Ohio legislature the past week, contemplating placing the privilege of practicing psychotherapy in the hands of the clergy of all denominations. This is but another onslaught on the Medical Practice Act. The cults and faddists believe that if they can only get the barriers down at any place, it would be a vantage point for further ingression. The Emmanuel movement was started at Worcester, Mass., with the laudable intent to offset Eddyism. It seems to have got beyond control of the originators, and this is one of the results of fanaticism and veiled cupidity. Every physician should write the member of the assembly from his district his absolute disapproval.—*Lancet-Clinic.*

